# Morphology, topology and dimensions of the heart and arteries of genetically normal and mutant mouse embryos at stages S21–S23

**DOI:** 10.1111/joa.12663

**Published:** 2017-08-03

**Authors:** Stefan H. Geyer, Lukas F. Reissig, Markus Hüsemann, Cordula Höfle, Robert Wilson, Fabrice Prin, Dorota Szumska, Antonella Galli, David J. Adams, Jacqui White, Timothy J. Mohun, Wolfgang J. Weninger

**Affiliations:** ^1^ Division of Anatomy & MIC Medical University of Vienna Vienna Austria; ^2^ The Francis Crick Institute London UK; ^3^ Wellcome Trust Centre for Human Genetics Oxford UK; ^4^ Wellcome Trust Sanger Institute Cambridge UK

**Keywords:** cardiovascular system, deciphering the mechanisms of developmental disorders, embryo, high resolution episcopic microscopy, mouse, mutant

## Abstract

Accurate identification of abnormalities in the mouse embryo depends not only on comparisons with appropriate, developmental stage‐matched controls, but also on an appreciation of the range of anatomical variation that can be expected during normal development. Here we present a morphological, topological and metric analysis of the heart and arteries of mouse embryos harvested on embryonic day (E)14.5, based on digital volume data of whole embryos analysed by high‐resolution episcopic microscopy (HREM). By comparing data from 206 genetically normal embryos, we have analysed the range and frequency of normal anatomical variations in the heart and major arteries across Theiler stages S21–S23. Using this, we have identified abnormalities in these structures among 298 embryos from mutant mouse lines carrying embryonic lethal gene mutations produced for the Deciphering the Mechanisms of Developmental Disorders (DMDD) programme. We present examples of both commonly occurring abnormal phenotypes and novel pathologies that most likely alter haemodynamics in these genetically altered mouse embryos. Our findings offer a reference baseline for identifying accurately abnormalities of the heart and arteries in embryos that have largely completed organogenesis.

## Introduction

Understanding embryo development remains a central challenge in biology. Not only does it underpin efforts to prevent or ameliorate the health burden of human developmental abnormalities, it also offers the opportunity to identify cellular and genetic interactions that circumscribe tissue differentiation, knowledge of which could guide efforts to develop novel, stem cell‐based therapies to treat human disease.

Among the animal models used for studying development, the mouse has proved of particular value. As a placental mammal, it shows the closest genetic and embryological similarity to humans and its genetic tractability enables the contribution of individual genes during development to be easily investigated. Identifying the impact of targeted gene mutation or ablation on normal embryogenesis forms the common starting point of such studies. In principle, this is a simple issue of comparative anatomy, in which any structural disparity between mutant and wild‐type embryos is identified. In practice, the accuracy and reliability of such comparisons can easily be confounded.

One obvious problem is that tissue arrangement and organ morphology undergo profound changes as embryos develop. Accurate phenotyping therefore requires the matching of developmental stages between experimental and control embryos. It is well established that embryos within a single litter can be at quite different developmental stages and, as a consequence, comparison with littermates or between embryos staged simply by harvest date (days *post coitum*) may not be appropriate. Even when anatomically defined staging criteria (such as the Theiler system) are employed, there are also periods of embryo development when rapid and major changes in the structure of particular organs may occur. As a result, accurate phenotyping depends upon more precise sub‐staging (Henkelman et al. 2016; Geyer et al. 2017b).

A second difficulty, which has so far received much less attention, is the extent to which normal embryo structure may show variability at any developmental sub‐stage, through either varying degrees in heterochrony or inherent variations in structure, morphology or topology of particular organs or tissues. With the advent of modern imaging modalities, it is now becoming possible to address this issue. Three‐dimensional imaging technologies now make it possible to examine embryo structure in sufficiently large numbers that population‐based variations can be identified.

Here we describe such a study, examining the heart and arteries of the mouse embryo. We have focused on the developmental window obtained by harvesting litters on the 15th day *post coitum* (E14.5), a period commonly used for phenotyping, as much of organogenesis has been completed by this time (Weninger et al. 2009; Mohun et al. 2013). Over 200 wild‐type C57BL/6N embryos form the basis of this study, generated as part of the screening programme ‘Deciphering the Mechanisms of Developmental Disorders’ (DMDD; https://dmdd.org.uk) (Mohun et al. 2013; Wilson et al. 2016a,b). DMDD uses high resolution episcopic microscopy (HREM) to study embryos carrying null mutations resulting in embryo lethality (Weninger et al. 2006; Weninger & Geyer, 2008; Geyer et al. 2009). This provides near‐histological quality image data that span the entire embryo and facilitate phenotyping using both 2D and 3D images (Weninger et al. 2014). By combining the resolution afforded by HREM with a simple but precise developmental sub‐staging system (Geyer et al. 2017b), 3D analysis of such a large number of embryos has enabled us to assess for the first time the range and frequency of anatomical variations affecting the heart and major arteries in normal embryos.

In addition, this population study of wild‐type embryos provides a reference baseline that facilitates phenotyping. We have used it to identify abnormalities in the heart and vessels among almost 300 homozygous mutant embryos from 55 embryonic or perinatal lethal mutant mouse lines. We present examples of commonly occurring abnormal phenotypes and describe novel pathologies revealed by HREM that may compromise survival by virtue of their impact on haemodynamics in these genetically altered mouse embryos.

## Materials and methods

We analysed the phenotype of a total of 474 E14.5 mouse embryos (298 mutants and 176 controls) bred from the C57BL/6N strain at the Wellcome Trust Sanger Institute (http://www.sanger.ac.uk/) as part of the DMDD project (https://dmdd.org.uk/) and its pilot (Mohun et al. 2013; Weninger et al. 2014). All embryos were carefully staged using forelimb maturation (Geyer et al. 2017b). As the number of controls of S21 and S22 was too small, we also included an additional 30 wild‐type embryos harvested at E13.5 and staged as S21 (25 embryos), S22‐ (4 embryos) and S22 (1 embryo).

Embryos were processed using the DMDD pipeline. They were fixed in Bouin's for at least 24 h, washed in phosphate‐buffered saline (PBS), dehydrated in an increasing series of methanol concentrations (10% increments with an additional step of 95%; 2 h for each step), and embedded in methacrylate resin (JB‐4, Polysciences) containing eosin and acridine orange (Weninger et al. 2006; Mohun & Weninger, 2012a). After polymerisation at room temperature, the resin blocks were baked at 90 °C for 1–2 days and used for HREM data generation (Mohun & Weninger, 2012b; Geyer et al. 2017a). Resulting digital datasets consisted of approximately 3000–4000 precisely aligned single images and had isotropic voxel sizes with edge lengths between 2.5 and 3.5 μm. All datasets are freely available at https://dmdd.org.uk/.

Following a specific protocol published recently, embryo datasets were analysed on MacPro computers (Weninger et al. 2014) operating a special routine created for the osirix ^®^ software (Wilson et al. 2016b). Detailed surface‐rendered 3D models of the arteries were generated from 16 embryos at S21–S23 (two to four controls per stage) and five mutants, on PC workstations using amira 5.4.5 (FEI Visualization Sciences Group).

**Figure 1:**
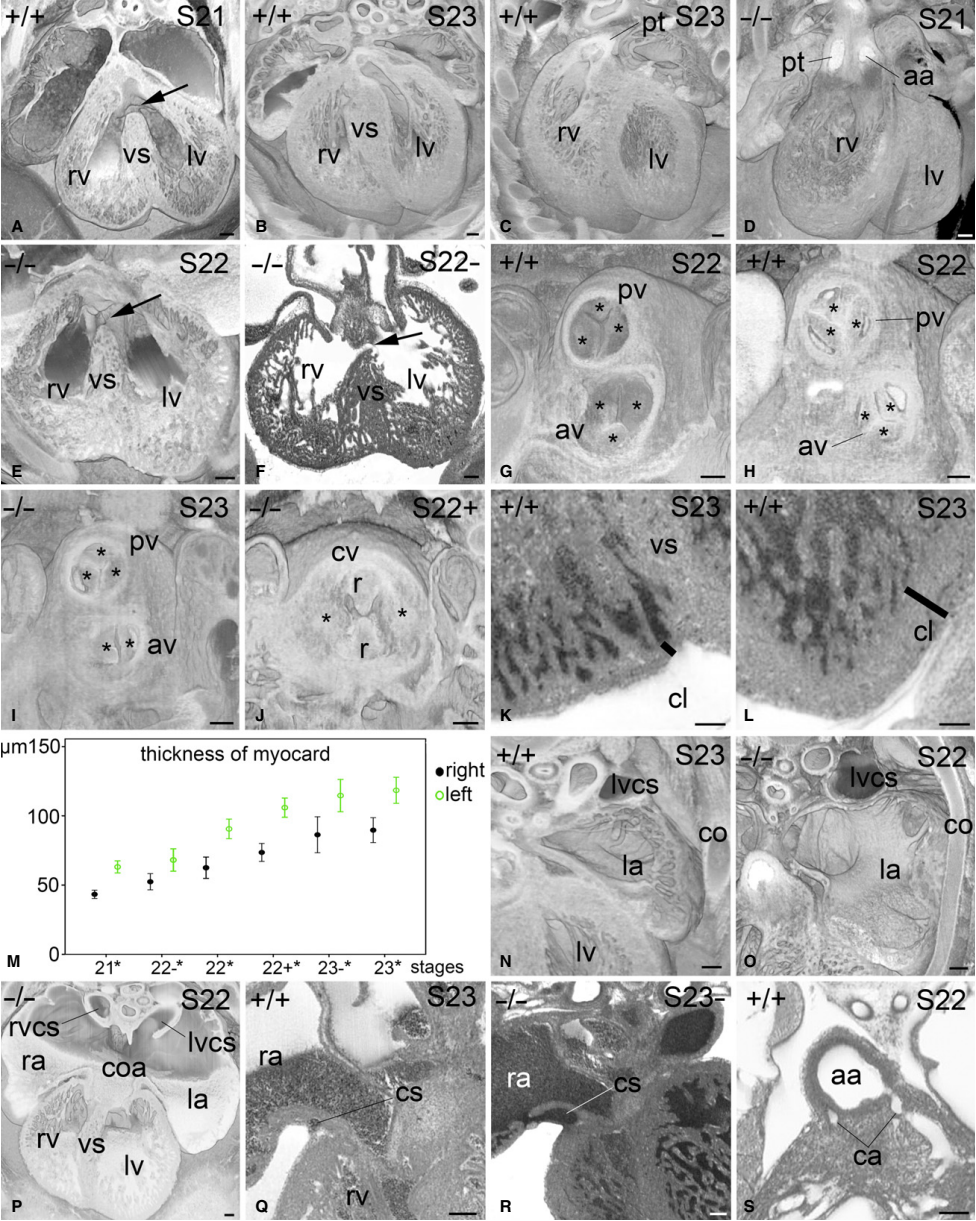
Heart of E14.5 mouse embryos. Bottom of all images is ventral. View is from cranial. (A–E) Virtually sectioned volume‐rendered heart models of genetically normal (A–C) and mutant (D,E) embryos of S21 and S23. Compared to the controls in (B and C), the mutant in (D) (Chtop^−/−^) shows double‐outlet right ventricle and the mutant in (E), (Atpa^−/−^), shows a perimembranous ventricular septal defect (arrow). Note that the perimembranous ventricular septal defect in the S22 embryo appears similar to the large interventricular communication and overriding aortic valve (arrow in A), which is normal for embryos of S21. (F) Atpa^−/−^ mutant with a muscular septal defect (arrow), view from cranial in an axial high‐resolution episcopic microscopy (HREM) image. (G–J) Volume‐rendered models of the semilunar valves. The cusps (asterisk) of normal S22 embryos can still appear as solid tissue protrusions (G) as in earlier stages or show cavitations (H), as in later embryos. Note the bicuspid aortic valve (av) in a Camsap^−/−^ mutant (I) and the common valve (cv) with still visible endocardial ridges (r) in an Ssr2^−/−^ mutant (J). (K,L) Compact layer of the myocardium in axial HREM sections. Note the thinness (black bar) of the compact layer of the right ventricle near the ventricle septum (vs) in (K). (M) Measurements that demonstrate gradual thickening of the compact myocardial layer with embryo maturation and differences in the thickness of the left‐ and right‐sided myocardium in genetically normal embryos. Significant differences in thickness are labelled with an asterisk. (N,O) Axially sectioned volume‐rendered models of the left atrial appendix with pectinate muscles. Note the large amount of muscle ridges in the control (N) and reduced numbers in an Atpa^−/−^ mutant (O). (P) Axially sectioned volume‐rendered model of an Rpgrip1l^−/−^ mutant showing common and largely symmetric atrium (coa). (Q,R) Axial HREM sections showing the right aspect of the coronary sinus (cs) in a control (Q) and a Cog6^−/−^ mutant. Note the connection of the coronary sinus to the right atrial appendix in the mutant. (S) Connection of the coronary arteries (ca) to the ascending aorta. aa, ascending aorta; av, aortic valve; ca, coronary artery; co, costa; coa, common atrium; cs, coronary sinus; cv, common valve; la, left atrium; lv, left ventricle; lvcs, left vena cava superior; pt, pulmonary trunk; pv, pulmonary valve; r, ridge; ra, right atrium; rv, right ventricle; rvcs, right vena cava superior. Scale bars: 100 μm.

The thickness of the compact layer of the myocardium was measured in axial HREM sections cutting through the region of the interventricular foramen. Three measurements were taken from each ventricle; one at its ventral wall, one at its lateral wall and the third halfway inbetween (Fig. 1A).

The position of the left subclavian artery was characterised using the 3D measurement tool in amira to measure the distance between the (proximal) branching point of the left subclavian artery and a virtual point set at the vessel wall of the descending aorta opposite the communication with the ductus arteriosus.

excel (Microsoft Office 2013 for Windows) and spss (IBM SPSS Statistics Version 22) were used for statistical analysis.

## Results

At the stages we studied, the principal features of fetal circulation and heart morphology are largely established. We focused our study on those features whose gradual maturation from S21 to S23 caused significant changes in morphology and topology. In addition, by analysing sufficient numbers, it became possible to assess the extent of morphological variation in cardiac and vascular anatomy among wild‐type embryos. These provided the basis for phenotyping of cardiovascular abnormalities in embryos from 54 of the 55 mutant lines analysed.

### Heart

Over the developmental stages analysed, the heart developed its definitive arrangement of chambers and tissues. However, as we have previously described, 97% of S21 embryos showed incomplete separation of the ventricular chambers, the size of the interventricular foramen gradually reducing in size with maturation (Wilson et al. 2016a; Geyer et al. 2017b). Even at S23, 2% of the S23 embryos showed a remnant of the interventricular foramen.

#### Valves

In S21 embryos, the aortic valve was arranged above this interventricular connection, almost resembling a pathologically overriding aortic valve. As development proceeded, it shifted more to the left, until it was strictly on the left side of the septum in S23 embryos. The pulmonary valve was arranged on the right, and more cranially, in all developmental stages of E14.5 (Fig. 1A–C). Examples of frequently observed malformations in mutants were double‐outlet right ventricle and perimembranous or muscular ventricular septal defects (Fig. 1D–F).

The semilunar valves were fully formed in all stages, but appeared as three solid tissue protrusions in embryos at stage S22 and younger, while showing characteristic cavitations that deepened with maturation in later embryos (Fig. 1G,H). Observed malformations in the mutants ranged from bicuspid aortic valve to common valves in embryos with persisting truncus arteriosus (Fig. 1I,J).

#### Myocardium

Measurements in axial sections on characteristic sites of the compact layer of the ventricle myocardium showed that its thickness doubles between S21 and S23. Interestingly, the ventral wall of the right ventricle near the interventricular septum was always considerably thinner than the wall of all the other heart segments (Fig. 1K–M). Furthermore, measurements taken from comparable sites at the ventral walls of the left and right ventricles showed that the compact layer of the left ventricle myocardium was significantly (*P* < 0.001) thicker than that of the right in each developmental (sub‐)stage (Fig. 1M).

#### Atrial chambers

In all embryos, the left and right atria were fully separated, with only the foramen ovale left as a connection. The appendices were large and contained pectinate muscles (Fig. 1N). Typical malformations in mutants were reduction of the number of pectinate muscles (Fig. 1O) and common atrium, the latter associated with malformations of the atrioventricular junction (Fig. 1P). The coronary sinus was formed in all embryos, connected to the left superior vena cava as it crossed under the lung veins and drained into the right atrium. Before entering the atrium, the sinus received large veins coming from the left ventricle myocardium. The distal end of the sinus was extended as an elongated and blindly ending sac rightwards, between the right atrial appendix and the right ventricle (Fig. 1Q). In eight of the 55 mutant lines, we detected a previously undescribed malformation, comprising an additional connection between the coronary sinus and the right atrial appendix (Fig. 1R).

#### Coronary ostia

Almost all embryos of S22 and older (80% of S22) and 26% of S21 showed a connection of both coronary arteries to the ascending aorta (Fig. 1S). Unambiguous identification of coronary ostia can be problematic with tissue shrinkage or with data from malformed hearts; nevertheless, at least a unilateral coronary artery was identified in 100% of embryos of S22 and older (93% of S22) and 77% of S21 embryos (Table 1).

**Table 1:** Frequency and mean dimensions of norm variations and morphological features in control embryos of stages 21–23

	21	22−	22	22+	23‐	23
Interventricular communication (%)	97	87	70	38	32	2
Myocard left (mean)	63 μm ± 2.13	68 μm ± 3.78	91 μm ± 3.47	106 μm ± 3.45	115 μm ± 5.75	119 μm ± 4.67
Myocard right (mean)	43 μm ± 1.46	52 μm ± 2.72	63 μm ± 3.77	74 μm ± 3.22	87 μm ± 6.41	90 μm ± 4.46
Coronary artery not connected (L/R) (%)	58/39	20/7	0/0	9/0	0/0	2/0
Distance subclavia – Ductus arteriosus (mean)	63 μm ± 8.23	122 μm ±7.85	177 μm ± 4.47	189 μm ± 3.54	207 μm ± 4.84	224 μm ± 3.99
Remnant of right 6th pharyngeal arch artery (%)	3.2	13.3	0	0	0	0
Remnant of right dorsal aorta (%)	90	93	50	53	32	18
Abnormal vertebral artery topology (%)	0	13	13	0	0	0
Enlarged blood vessels in wall of aorta (%)	9.7	26.7	13.3	44.7	34.2	24.4
Blood‐filled vessels between pericard and diaphragm (%)	6.5	20	33.3	29.8	31.6	28.9
Blood‐filled capillaries in ovary (%)	14.3	16.7	50	35.2	22.2	15.4
Blood‐filled capillaries in brain (%)	55	47	23	38	32	40

### Arteries

**Figure 2:**
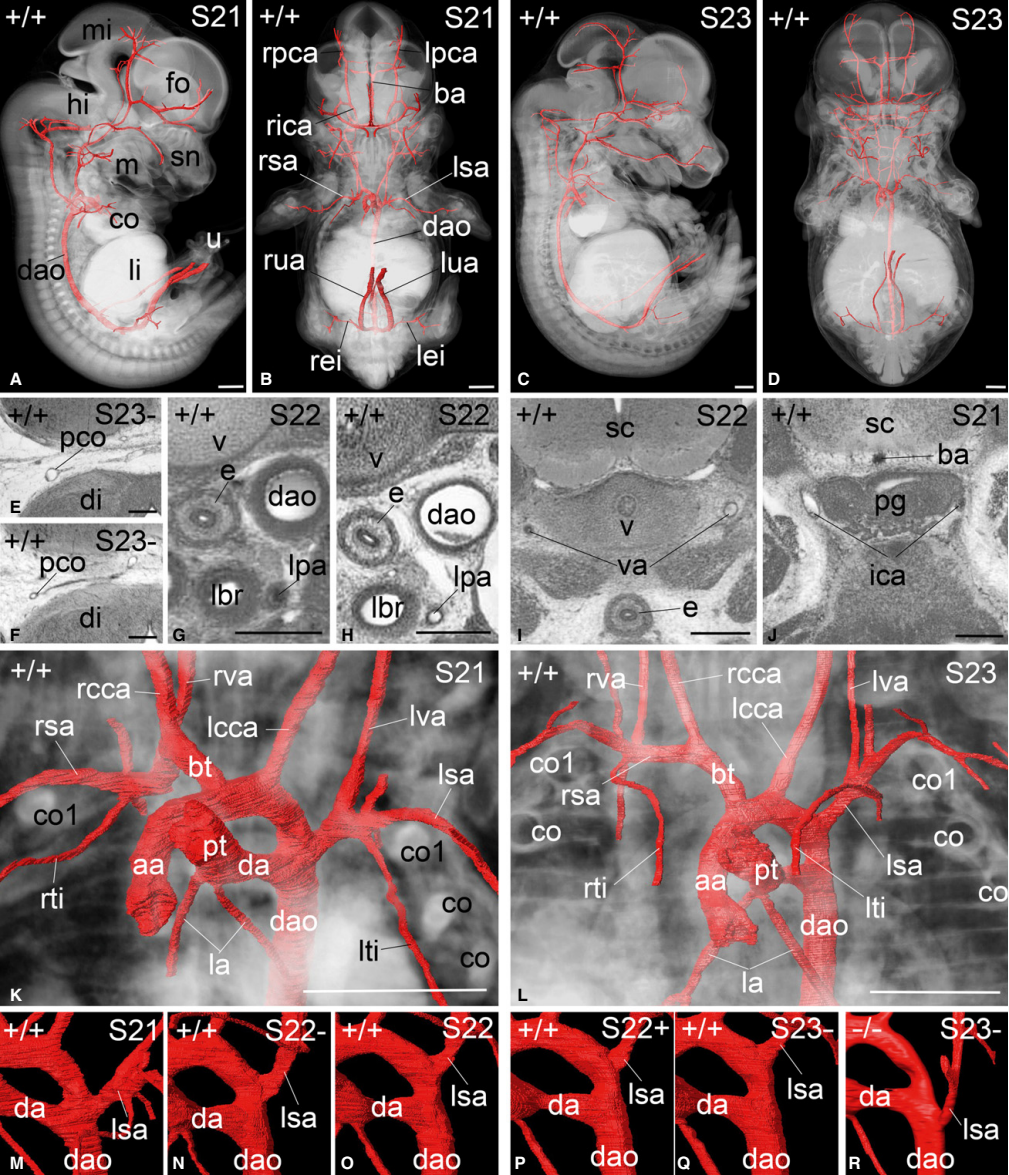
Arteries of E14.5 mouse embryos. (A–D) Combined volume‐rendered models of whole embryos and surface‐rendered models of large arteries at S21 (A,B) and S23 (C,D) from the right (A, C) and ventrally (B, D). (E–J) Axial high‐resolution episcopic microscopy (HREM) sections, view from cranial. Bottom of images is ventral. Compare (E) and (F) for the variable diameter of the posterior communicating artery, (G) and (H) for the variable appearance of the wall thickness of the descending aorta (dao), and the contralateral arteries in (I) and (J) for the variable appearance of the vertebral (va) and parasellar segments of the internal carotid arteries (ica). (K,L) Volume‐rendered models of the thorax and surface‐rendered models of the great intra‐thoracic arteries at S21 (K) and S23 (L) ventrally. Bottom of images is caudal. Note the position of the lvcs ( left vena cava superior) arteries in respect to the 1st rib (co1). Due to tissue maturation, rib material appears white at S21 and black at S23. (M–R) Surface‐rendered models of the great intra‐thoracic arteries ventrally, showing the origin of the left subclavian artery (lsa) with respect to the ductus arterious (da). View as in (I) and (J). The distance between da and lsa steadily increases during maturation (M–Q). Note the heterochronic origin of the subclavian artery from the descending aorta (dao) in the Adamts3^−/−^ mutant shown in (R). aa, ascending aorta; ba, basilar artery; bt, brachiocephalic trunk; c, cor; co, costa; da, ductus arteriosus; dao, descending aorta; di, diencephalon; e, oesophagus; fo, forebrain; hi, hindbrain; ica, internal carotid artery; la, lung artery; lbr, left bronchus; lcca, left common carotid artery; lei, left external iliac artery; li, liver; lpa, left pulmonary artery; lpca, left posterior cerebral artery; lsa, left subclavian artery; lti, left internal thoracic artery; lua, left umbilical artery; lva, left vertebral artery; m, Meckel's cartilage; mi, midbrain; pg, pituitary gland; pco, posterior communicating artery; pt, pulmonary trunk; rbr, right bronchus; rica, right internal carotid artery; rcca, right common carotid artery; rei, right external iliac artery; rpca, right posterior cerebral artery; rsa, right subclavian artery; rti, right internal thoracic artery; rua, right umbilical artery; rva, right vertebral artery; sn, snout; u, umbilical hernia; v, vertebra; va, vertebral artery. Scale bars: 500 μm (A–D, K–R), 200 μm (E–J).

All major arteries were already formed in embryos at stages S21–S23. In general, vessels could be followed further distally in later embryos than in their younger counterparts. Strikingly, the lumen diameters did not increase proportionally with whole embryo size during maturation and, as a result, vessels such as the aorta often appeared larger in early embryos than in older embryos (Fig. 2A–D). All arteries showed remarkable differences in lumen dimensions and wall thicknesses (Fig. 2E,F). This was especially true for left‐ and right‐sided arteries, but also for subsequent segments of the same vessel along its course (Fig. 2G,H). Artery lumens varied in appearance between embryos. They were either free of blood and clearly demarcated from the blood vessel wall, or partially or completely occluded by blood cells, which blurred the border between lumen and wall (Fig. 2E–H).

#### Great intrathoracic arteries

The pulmonary trunk emerged from the right ventricle and took an almost horizontal, dorsally directed course. In approximately two‐thirds of the embryos, it gave rise directly to two narrow pulmonary arteries; in the remainder it formed a short narrow trunk, which then bifurcated into the two lung arteries. The arterial duct was a continuation of the pulmonary trunk, having almost the same lumen diameter and joining the aorta at the transition of aortic arch to descending aorta (Fig. 2I,J).

The aorta emerged from the left ventricle, ascended and continued into the aortic arch. It gave rise to the brachiocephalic trunk, always dividing into right common carotid and right subclavian, left common carotid and left subclavian arteries. The aortic arch was positioned at the level of the superior thoracic aperture at S21, but its position gradually descended towards the first intercostal by S23‐ (Fig. 2I,J). This was accompanied by a gradual increase in length of the proximal segments of the subclavian arteries.

In 99% of the wild‐type embryos, the origin of the left subclavian artery was proximal to the connection of arterial duct and aorta. In two S21 embryos, its origin was instead at the level of the connection, or slightly distal to it. The distance between the connection of arterial duct and the origin of the left subclavian artery increased throughout the period from S21 to S23 (Table 1, Fig. 2K–P).

Of the genetically normal embryos, 1.5% had a strand‐like connection without visible lumen between the arterial duct and the right subclavian artery, which we identified as a remnant of the right sixth pharyngeal arch artery. An additional 50% had a similar strand‐like connection between the subclavian artery and the descending aorta crossing from right to left dorsal to the oesophagus. We identified this as a remnant of the right dorsal aorta. In 1.9%, this connection retained a lumen. The frequency of both decreased with maturation (Table 1). One embryo showed a double‐lumen aortic arch.

#### Head arteries

##### Internal carotid arteries

**Figure 3:**
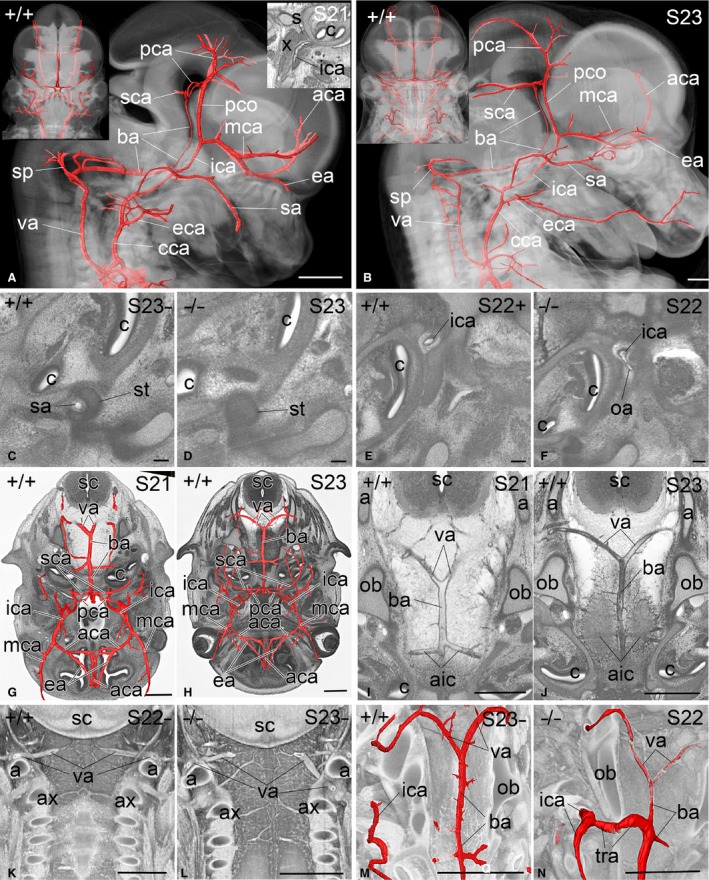
Head arteries of E14.5 mouse embryos. (A,B) Volume‐rendered models of the head and surface‐rendered models of its arteries at S21 (A) and S23 (B) from right. Bottom of panel is caudal. Frontal view in top left insert. Top right insert in (A) holds sagittal high‐resolution episcopic microscopy (HREM) section from right that demonstrates the position of the internal carotid artery (ica) with respect to cochlea (c), inferior vagus ganglion and vagus nerve (X) and superior cervical ganglion (s). (C–F) Axial HREM sections through the head. Bottom of image is medial, right of image is ventral. Compare (C) and (D) for the normal position of the stapedial artery (sa) running through the anlage of the stapes in genetically normal embryos and the absence of the sa in an Adcy9^−/−^ mutant. Note the abnormal shape of the stapes in (D). A dorsal ophthalmic artery (oa) originates from the internal carotid artery (ica) in a Nsun2^−/−^ mutant (F). (G,H) Surface‐rendered models of the head arteries above superimposed on HREM sections showing the circulus arteriosus and its tributaries cranially at S21 (G) and S23 (H). Bottom of image is ventral. (I,J) Axial HREM sections showing the formation and course of the basilar artery (ba) at S21 (I) and S23 (J). View as in (G) and (H). (K,L) Coronally sectioned volume‐rendered models of the cervical spinal canal from ventral view. Bottom of image is caudal. Note the abnormal position of the left vertebral artery (va) below the posterior arch of the atlas (a) in a Pdzk1^−/−^ mutant (L). (M,N) Obliquely sectioned volume‐rendered models of the central head region and surface‐rendered models of its arteries. View as in (G–J). The internal carotid (ica) and basilar arteries (ba) of the Sh3pxd2a^−/−^ embryo displayed in (N) are connected by a persisting trigeminal artery (tra). Note the diameter of the ba segments proximal and distal to this connection. a, atlas; aic, anterior inferior cerebellar artery; ax, axis; cca, common carotid artery; eca, external carotid artery; ica, internal carotid artery; aca, anterior cerebral artery; ba, basilar artery; c, cochlea; eca; external carotid artery; ea, ethmoidal artery; mca, middle cerebral artery; oa, ophthalmic artery; ob, occipital bone; pca, posterior cerebral artery; pco, posterior communicating artery; s, superior cervical ganglion; sa, stapedial artery; sc, spinal chord; sca, superior cerebellar artery; sp, spinal artery; st, stapes; va, vertebral artery; x, vagus nerve. Scale bars: 500 μm (A, B, G–N), 100 μm (C–F).

The common carotid arteries ascended symmetrically in the upper thorax and neck, forking at the level of the tongue and thyroid cartilage to form the external (ECA) and internal carotid arteries (ICA). The latter ascended symmetrically without branching until they reached the skull base. Along the entire neck, both the common carotid and subsequently the internal carotid arteries were accompanied by the vagus nerve, jugular vein and sympathetic trunk. Below the otic vesicle, the internal carotid arteries lay between the (sympathetic) superior cervical and inferior vagal ganglia (Fig. 3A,B). Here they gave rise to the stapedial arteries, which branched laterally at an angle of approximately 90°, and after a short distance turned ventrally to pass through the anlage of the stapes (Fig. 3C). In their further course, the stapedial arteries gave rise to the medial meningeal arteries and continued into the orbit, where they split into their final branches. In some mutants the stapedial artery was found to be absent inside the stapes (Fig. 3D).

After releasing the stapedial arteries, the ICAs ascended medially to the cochlea, straight through the forming carotid canal and through the parasellar region (Fig. 3E), leaving the pituitary gland medially (Fig. 2H) and the trochlear nerve laterally. Some mutants showed an artery arising from the intracanalicular or parasellar ICA segment and heading for the orbit (Fig. 3F). After leaving the parasellar region, the ICAs turned anteriorly and split into the anterior and medial cerebral arteries. Before splitting, at their turning points, the ICAs gave rise to the posterior communicating arteries. These arteries ascended in a slightly dorsal direction and were joined by posterior cerebral arteries, which were often narrow vessels and sometimes barely visible from routine HREM data. In almost all embryos examined, the arteries that continued from the joining point were of the same diameter as the posterior communicating arteries, which therefore appeared to be the main source of blood supply to the occipital parts of the forebrain (Fig. 3A,B,G,H).

The medial cerebral arteries ran straight towards the region of the forming lateral fossae of the telencephalon, whereas the anterior cerebral arteries took a medio‐frontal course. Each anterior cerebral artery gave rise to a rather thick ethmoidal artery, which entered the nasal cavity. The left and right anterior cerebral arteries joined and formed a single vessel, which turned upwards to run between the forming hemispheres of the telencephalon (Fig. 3G,H). Joining of the anterior cerebral arteries was variable, and quite often there were additional connections between the arteries.

##### Vertebral arteries

The vertebral arteries arose from the intrathoracic portions of the subclavian arteries and ran towards the 6th cervical vertebra, where they ascended the canal formed by the foramina of the lateral processes of the cervical vertebrae. During their passage through the foramina of the transversal processes, they gave rise to spinal branches of variable diameters, which segmentally entered the spinal canal (Fig. 3B). These vessels were not clearly visible in all routine datasets. After passing through the foramina of the lateral processes of the atlas, the vessels turned medially and entered the spinal canal between the forming atlas and occipital bone (Fig. 3G–K). Inside the spinal canal the vertebral arteries gave rise to the anterior spinal arteries, which ran towards the spinal chord, and joined and formed a single vessel that descended between the spinal chord and the bodies of the cervical vertebrae.

The vertebral arteries then ran towards the midline and joined to form the basilar artery (Fig. 3G–J), entering the spinal column cranial to the atlas. In six embryos (2.9%) no such arrangement was detected. Instead, the segmental branch, entering between atlas and axis, replaced the vertebral artery and joined the contralateral vertebral artery to form the basilar artery (Fig. 3L). In five of these six embryos there was no artery between atlas and occipital bone; in one embryo, an artery entered but solely fed the anterior spinal artery. In mutants, this variation occurred in a much higher proportion (16%).

##### Basilar artery

The basilar artery ran ventrally to the pons in a rostral direction within the medio‐sagittal plane until it reached the acute mesencephalic flexure. Here it split into the left and right superior cerebellar arteries (Fig. 3A,B). Each superior cerebellar artery immediately gave rise to a very narrow, sometimes barely visible posterior cerebral artery that ran laterally and joined the usually much larger, ipsilateral posterior communicating artery (Fig. 3G,H). In 5.8% of the wild‐type embryos, this segment of the posterior cerebral artery was missing unilaterally. In a single embryo (0.5%), the right posterior communicating artery was missing.

Along its course, the basilar artery gave rise to a number of small arteries and formed two large vessels, the anterior inferior cerebellar arteries, which circumvent the brainstem at the level of the inner ear to reach the forming cerebellum (Fig. 3G,H). From their lateralmost parts, small labyrinthine arteries arose to enter the inner ear. A common variation was the unilateral presence of an additional large cerebellar artery that arose distal to one of the anterior inferior cerebellar arteries. A relatively common abnormality in mutant embryos was the presence of an additional connection between the internal carotid artery (through its extracranial, intracanalicular or parasellar segment) and the basilar artery at the level of the origin of the inferior cerebellar arteries. Usually this connection was the main source of blood supply to the brain distal to this point (Fig. 3M,N).

##### External carotid arteries

**Figure 4:**
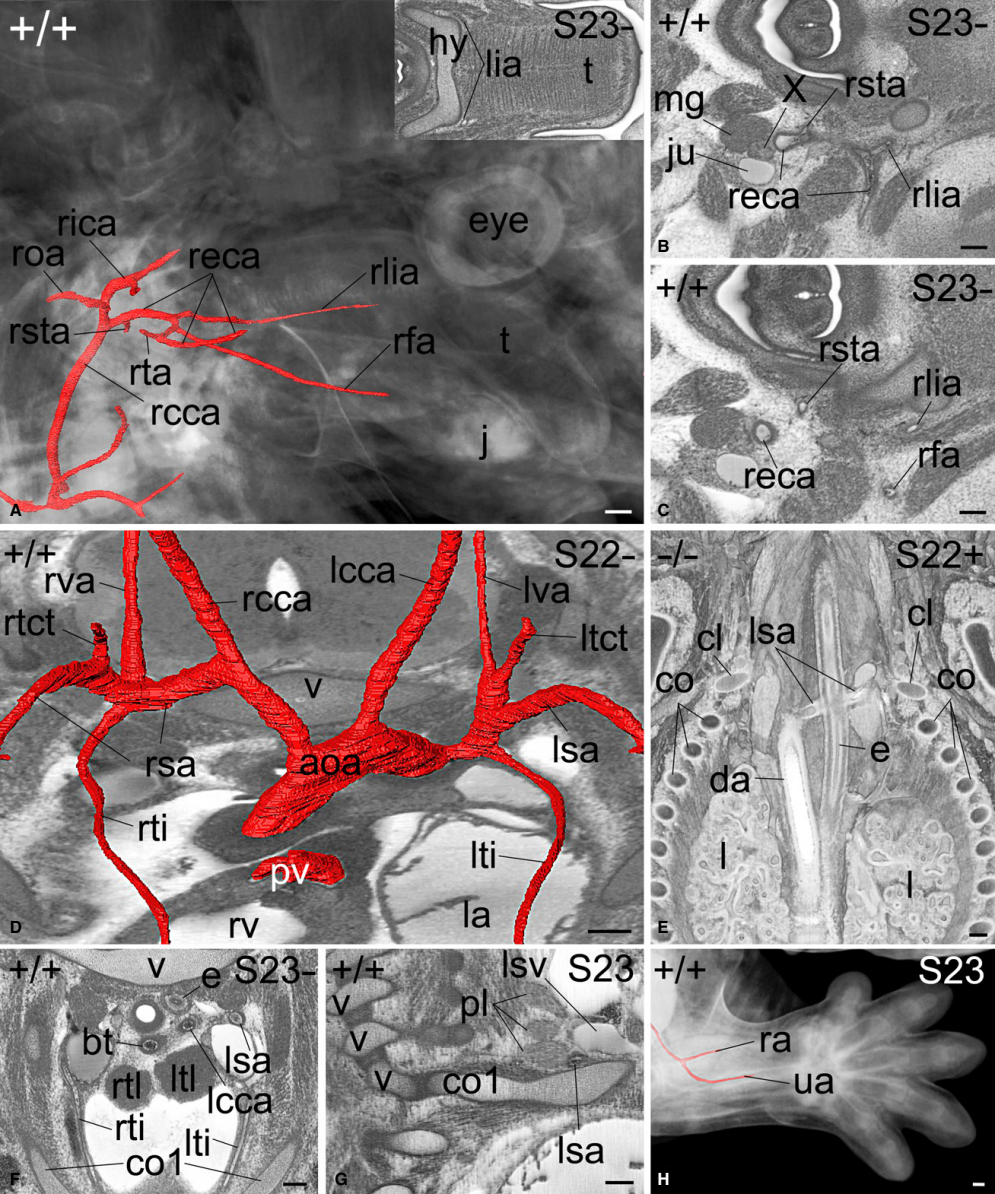
External carotid (A–C) and subclavian arteries (D–H) and branches. (A) Volume‐rendered model of the head and surface‐rendered model of the external carotid artery and branches in a S23‐ embryo from the right. Bottom of image is caudal. Insert shows an axial section through the tongue (t) with entering lingual arteries (lia). (B,C) Axial high‐resolution episcopic microscopy (HREM) sections showing the right sided external carotid artery (reca) and branches viewed from cranial. Right side of image is ventral. (D) Surface‐rendered model of the aortic arch and great intrathoracic arteries superimposed on an axial HREM section from ventro‐cranial. Bottom of image is ventral. Note the origin of the intrathoracic branches of the left (lsa) and right (rsa) subclavian arteries. (E) Coronally sectioned, volume‐rendered model of the thorax of a 4933434E20Rik^−/−^ embryo from ventral. Bottom of image is caudal. Note the left subclavian artery (lsa) that originates from a right‐sided descending aorta and crosses dorsal to the oesophagus (E) towards the left 1st rib (left retroesophageal subclavian artery). (F) Course of the internal thoracic arteries (lti, rti) towards and along the 1st rib (co1) in an axial HREM section viewed from cranial. Bottom of image is ventral. (G) Sagittal HREM section through the cervico‐thoracal junction of a S23 embryo. Bottom of image is caudal. Note the topology of the brachial plexus (pl), lsa and left subclavian vein (lsv). (H) Volume‐rendered model of the left forelimb and surface‐rendered model of radial (ra) und ulnar (ua) arteries from lateral. aoa, aortic arch; bt, brachiocephalic trunk; c, costa; cl, clavicula; da, descending aorta; e, oesophagus; lcca, left common carotid artery; ltct, left thyrocervical trunk; lsa, left subclavian artery; lti, left internal thoracic artery; ltl, left thymic lobe; lva, left vertebral artery; rcca, right common carotid artery; rea, left retroesophageal subclavian artery; reca, right external carotid artery; rfa, right facial artery; rica, right internal carotid artery; rlia, right lingual artery; roa, right occipital artery; rsa, right subclavian artery; rsta, right superior thyroid artery; rtct, right thyrocervical trunk; rti, right internal thoracic artery; rva, right vertebral artery; rtl, right thymic lobe. Scale bars: 100 μm.

The external carotid arteries arose from the common carotid arteries and could be tracked until they reached Meckel's cartilage near and below its connection to the forming otic ossicles. Immediately after its origin, the external carotid artery gave rise to the lingual, facial, superior thyroid and, sometimes, occipital arteries. In 49% the right and in 72% the left occipital artery arose from the internal carotid artery. The lingual arteries could be tracked towards and inside the muscles of the tongue. The facial arteries could be tracked towards the lower jaw. In embryos at S22‐ and older, the superior thyroid artery could also be tracked towards its perforation of the thyroid cartilage (Fig. 4A–C). Identification of other branches of the external carotid was not always possible in routine HREM data, although the superficial temporal artery was usually detected in embryos by stage S23‐. In one of the wild‐type embryos, no left‐sided common carotid artery existed. Instead, the left internal and external carotid arteries directly arose from the aortic arch (in humans this is accepted as a rare norm variant).

##### Subclavian artery

The right subclavian artery arose from the brachiocephalic trunk, whereas the left one originated from the aortic arch (Fig. 4D). In mutants with *situs inversus totalis* or *situs ambiguous* associated with inverse situs of the great intrathoracic arteries, peculiar abnormalities such as left retroesophageal subclavian artery (left‐sided A. lusoria; Fig. 4E), or origin of the left subclavian artery from a left‐sided brachiocephalic trunk, could be identified.

Inside the thorax, each subclavian artery gave rise to a clearly visible vertebral artery, (thyro)‐cervical trunk and internal thoracic artery, which followed the inside of the first rib towards the internal side of the ventral chest wall (Fig. 4F). In some of the older embryos the superior intercostal and deep cervical arteries could also be identified. Each subclavian artery then was joined by the subclavian vein ventrally and the brachial plexus dorsally, before the three of them passed the first rib cranial and dorsal to the scalenic tubercle (Fig. 4G). The continuation of the subclavian artery (axillar and then brachial artery) travelled into the arm and could be tracked until it reached the elbow, where the radial and ulnar arteries were formed (Fig. 4H).

#### Arteries of the lower body

**Figure 5:**
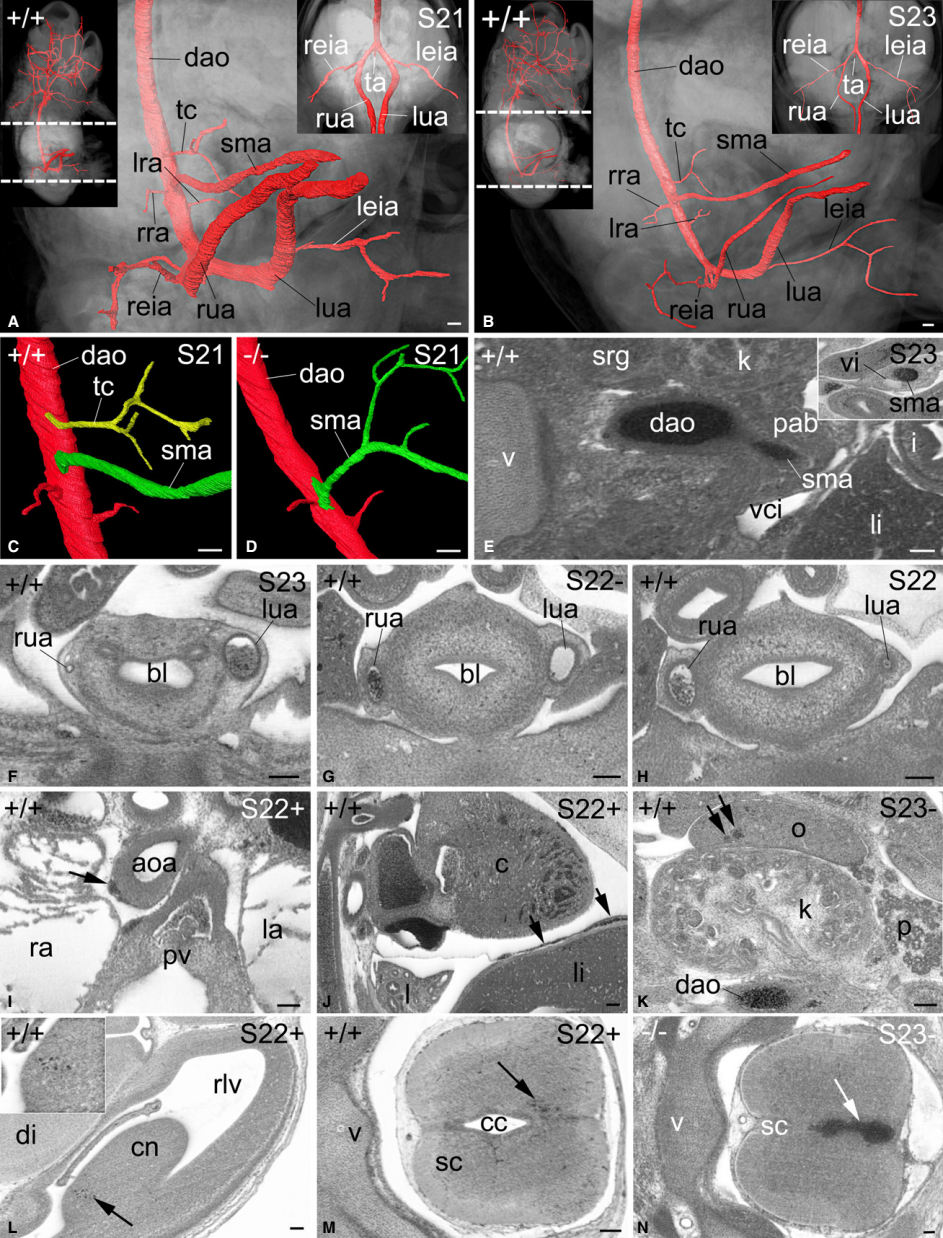
Arteries of the lower body of E14.5 mouse embryos. (A, B) Volume‐rendered models of the lower body and surface‐rendered models of its arteries at S21 (A) and S23 (B) from right. Insert to the right show a ventral and slightly cranial view. Inserts to the left show the entire embryo from anterolateral view. (C,D) Surface‐rendered models of the descending aorta (dao), coeliac trunk (tc) and superior mesenteric artery (sma) from the right in a genetically normal (C) and an H13^−/−^ mutant (D). The coeliac trunk is missing in the mutant. (E) Right‐sided origin of the superior mesenteric artery (sma). Axial high‐resolution episcopic microscopy (HREM) section. Right side of image is ventral. Note the relation to the arteries to the paraaortic bodies (pab). Insert shows sma and vitelline vein (vi) in the covering of the umbilical hernia. Compare the diameters of the sma in (E) and the insert. (F–H) Umbilical arteries in axial HREM sections, cranial view. Bottom of image is ventral. Note the diameters of the left (lua) and right (rua) umblical arteries. (I) Ectatic vasa vasorum in the wall of the ascending aorta (aoa) in an axial HREM section, cranial view. Bottom of image is ventral. (J) Ectatic blood vessels (arrow) between pericardium and diaphragm in a sagittal HREM section viewed from right. Right of panel is ventral. (K–M) Ectatic, blood‐filled capillaries (arrows) in right ovary (K), caudate nucleus (cn in L) and spinal chord (sc in M). Axial HREM sections, cranial view. Right side of panels is ventral. (N) Haemorrhage in tissues and central canal of the sc in an Rala^−/−^ mutant. Axial HREM section, cranial view. Right side of image is ventral. aoa, ascending aorta; bl, bladder; c, cor; cc, central canal; cn, caudate nucleus; dao, descending aorta; di, diencephalon; i, intestine; k, kidney; l, lung; la, left atrium; leia, left external iliac artery; li, liver; lra, left renal artery; lua, left umbilical artery; o, ovary; p, pancreas; pab, paraaortic body; pv, pulmonary valve; ra, right atrium; reia, right external iliac artery; rlv, right lateral ventricle; rra, right renal artery; rua, right umbilical artery; sc, spinal cord; sma, superior mesenteric artery; srg, suprarenal gland; ta, tail artery; tc, coeliac trunk; v, vertebra; vci, vena cava inferior. Scale bars: 100 μm.

The descending aorta took its course inside the thorax and lower body ventrally and slightly left of the vertebral bodies (Fig. 2A–D). It entered the retroperitoneum by squeezing through the muscle fibres of the left lumbar crus of the diaphragm and continued until it reached the level of the 4th lumbar vertebra. Here it split into the left and right common iliac arteries and a small tail artery, which represented its direct continuation (Fig. 5A,B). Along its way, the descending aorta segmentally released paired spinal arteries, which were often too small to be followed towards the intervertebral foramina and into the spinal canal.

##### Intestinal arteries

The first abdominal branches of the descending aorta were the coeliac trunk and the superior mesenteric artery. Both arose from the right aspect of the blood vessel circumference (Fig. 5C–E). The coeliac trunk ran ventrally and split into two clearly discernible arteries along with a third, very small and sometimes indistinct artery. These ran towards the liver, stomach and spleen, but in none of the stages examined could the arteries be fully tracked to their final target.

The superior mesenteric artery passed through the material of the para‐aortic bodies and met the superior mesenteric vein. Together they ascended directly towards the umbilical region, where they entered the umbilical hernia, running between the jejunum sling leaving the embryo body and colon entering it. Inside the hernia the artery gave rise to numerous small branches running to the intestinal slings. The artery itself, however, continued towards the right‐sided wall of the covering of the umbilical hernia, where it joined the remaining vitelline vein. In a proportion of embryos from S22+, S23‐ and S23 stages, the intestine had already rotated and the artery ran to the now left‐sided wall of the umbilical hernia. Interestingly, the diameter of the segment of the superior mesenteric artery running in the covering of the umbilical hernia was roughly the same as the diameter of its main stem (Fig. 5E).

##### Renal arteries

Distal to the origin of the mesenteric artery, the renal arteries and a number of small arteries that could only be tracked for a short distance arose from the left and right aspects of the aorta (Fig. 5A,B).

##### Iliac arteries

In all embryos, the left and right common iliac arteries split into external iliac and internal iliac arteries. The external iliac arteries were very narrow and continued into the femoral artery, which could be tracked to the proximal hindlimb. Occasionally a very thin ischiadic artery arose from the branching point of external and internal iliac arteries and also entered the hindlimb.

The internal iliac arteries were of roughly the same diameter as the common iliac arteries and had no other clearly visible branches other than the umbilical arteries. Consequently, these arteries had largely the same diameter as the ipsilateral internal iliac and common iliac arteries. In 33% of the wild‐type embryos examined, the left and right umbilical arteries were of roughly the same diameter; in 46%, the diameter of the right‐sided umbilical artery was larger than that of the left‐sided one; in the remaining 21%, the left artery was larger than the right (Fig. 5F–H). In many embryos one of the arteries was extremely thin, with barely any lumen visible.

#### Capillaries

Twenty‐seven percent of the embryos showed significantly enlarged capillary‐sized vessels in the outer parts of the right wall of the ascending aorta where it continues into the aortic arch (Fig. 5I). All embryos showed prominent, blood‐filled vessels between the pericardium and diaphragm, these ranging from mildly ectatic (Fig. 5J) to extremely enlarged. Clusters of enlarged capillary‐sized vessels also occurred in other regions of wild‐type embryos. For example, 21% of females possessed enlarged, blood‐filled capillaries in their ovaries (Fig. 5K) and 38% of all embryos showed such vessels inside the developing brain and spinal chord (Fig. 5L,M). None of these features showed any stage preference. In mutant embryos, malformations resembling such clusters of ectatic vessels included spinal haemorrhage (Fig. 5N) or haemangioma.

## Discussion

Adult and fetal mouse anatomy, especially the major components of the cardiovascular system, is well documented in reference texts (Bugge, 1970; Theiler, 1989; Kaufman, 1992). These long‐standing accounts distilled knowledge from a combination of anatomical observation and histological studies to provide what has proved to be relatively accurate and enduring descriptions, underpinning decades of subsequent research. Their authors were not, however, able to benefit from the insights afforded by the development of 3D imaging procedures and, with notable exceptions (Baldock et al. 2015), such standard descriptions remain unaltered.

For mouse embryos, the advent of novel imaging procedures has had a dramatic impact on our appreciation of the changing structure and topology of organs and tissues as they develop and mature prior to term. This is not because of improved image resolution of such structures, as no modern imaging modality has as yet improved on the rich detail captured by conventional histology, with all its varied staining procedures. Instead, new imaging methods such as OPT, μCT and HREM offer the ability to visualise embryo anatomy in 3D and the feasibility of analysing large numbers (Norris et al. 2013). Three‐dimensional visualisation allows an appreciation of topology and morphology that is, at best, challenging to achieve with histological studies. The nature of the data lends itself to novel forms of computational analysis that permits accurate quantitation (Wong et al. 2014, 2015) and comparison of individual embryos in a manner impossible with histology (Henkelman et al. 2016).

Crucially, both such approaches can be applied in studies with tens or hundreds of embryos and it is this ability to study populations rather than individuals that can be transformative. Rather than examining the anatomy at a relatively few, defined developmental stages, with sufficient numbers it is possible to approach a description of the actual developmental continuum (Wong et al. 2015), accurately capturing rapid changes and transitory features. In addition, studies on such a scale can for the first time determine the range of variation in anatomical structures that occurs in a normal population and can also allow abnormalities showing only low penetrance to be identified.

For the most part, such studies have yet to be undertaken. A few selected cardiovascular structures have been subjected to detailed 3D analysis and quantitation (Hiruma et al. 2002; Berrios‐Otero et al. 2009; Weninger et al. 2009; Geyer & Weninger, 2012, 2013; Geyer et al. 2012; Captur et al. 2016). Similarly, some progress has also been made in re‐examining morphogenesis of the developing heart chambers and their great vessels (Anderson et al. 2012, 2014, 2016a,b; Spicer et al. 2014; Captur et al. 2016). Our study is the first effort to use 3D analysis to establish an accurate reference description of the heart and arterial vessels of E14.5 embryos. Identifying the normal structures, their variability and their changes during this developmental window proved a necessary precondition for phenotyping genetically abnormal embryos in the DMDD programme (https://dmdd.org.uk) (Mohun et al. 2013; Wilson et al. 2016a). Our data is for the widely used CB57BL/6N strain and although it is likely to be very similar in other genetic backgrounds, these will require individual study to identify strain‐specific variations (see for example Captur et al. 2016).

With the resolution afforded by HREM, it is possible to combine examination of whole organ morphology with detailed examination of blood vessel topology, and tracking of even the smallest arteries was possible in most of the examined datasets of control embryos. In some, either poor image quality or the presence of blood in the vessel lumen hindered tracking of small vessel branches and our descriptions and statistics are limited to those structures we could unambiguously identify in all datasets. We were not, for example, able to analyse the coronary arteries or the arrangement of their ostia despite their importance, although this is feasible with exsanguination of isolated hearts (Captur et al. 2016).

Precise staging of embryos is essential for accurate identification of malformations (Geyer et al. 2017b) and this is exemplified by our studies of the E14.5 heart. In early E14.5 embryos, the presence of a significant interventricular foramen which closes by late E14.5 can be confused with a perimembranous ventricular septal defect. Similarly, the compact layer of the myocardium increases significantly in thickness during the E14.5 window and, without accurate sub‐staging, ‘thin myocardium’ might be diagnosed incorrectly in early samples. Our studies also show the importance of choosing the appropriate myocardial segment for measurement. The myocardium of the antero‐septal part of the right ventricle is, for example, constantly thinner than that of other parts of the ventricle myocardium.

Our data also indicate a number of difficulties that impede identification of some important anatomical abnormalities. We found that wall thickness and lumen diameters of blood vessels show dramatic inter‐ and intra‐embryonic variability, seriously hindering reliable diagnosis of blood vessel stenosis. These findings are consistent with variability in dimensions of the large intrathoracic arteries of mouse embryos previously revealed by metric analysis (Weninger et al. 2009). Stenosis and dilation can only therefore be scored if variation significantly exceeds the considerable range of normal dimensions. Our HREM data highlight an additional and hitherto largely unrecognised difficulty in the diagnosis of telangiectasia, tissue haemorrhages and haemangioma. A large number of genetically normal embryos show enlarged capillaries in delimited areas of the neural and ovarian tissues that could be confused with haemorrhages. In addition, enlarged blood vessels in the space between diaphragm and pericardium and the transition of the ascending aorta to the aortic arch, could be misdiagnosed as haemangioma.

Although our data cover the E14.5 developmental period, because of the variability of developmental stage within litters, our descriptions will also be appropriate for a proportion of embryos harvested a day later, at E15.5. This overlap is recognised within the Theiler staging system, E14.5 holding Theiler stages TS22 and TS23, and E15.5 encompassing TS23 and TS24 (Theiler, 1989; Kaufman, 1992). It is likely to have added relevance in the light of our finding that development is often slower in genetic mutants than in control littermates (Geyer et al. 2017b). A significant proportion of mutants harvested at E15.5 might therefore require comparison with the normal E14.5 embryos we have described.

Although the focus of our study was the analysis of normal development, its goal was to define a baseline for accurate phenotyping of abnormalities in mutant embryos. The impact such data will have on precise phenotype interpretation in mouse embryos with specific gene mutations (Sucov et al. 1994; Lin et al. 1997; Tanaka et al. 1999; Srivastava & Olson, 2000; Weninger et al. 2005) and on large‐scale phenotyping programmes is evident. We have attempted to illustrate this by presenting selected, unusual malformations detected in the heart and arteries of genetic mutants studied in the DMDD programme and which are best understood on the basis of a detailed developmental baseline. Examples are the connections between the basilar and internal carotid arteries, which we interpret as persistent segments of the trigeminal artery, and branches arising from the parasellar internal carotid artery, which we identify as the persisting dorsal ophthalmic artery. The latter is frequently associated with the absence of the segment of the stapedial artery that passes through the stapes. Our data have also revealed intriguing, and to our knowledge as yet unrecognised, abnormalities such as the connection of the coronary sinus to both the right atrium and the appendix of the left atrium. This represents a novel shunt between the left‐ and right‐sided heart at the level of the atria and is likely to have a significant haemodynamic impact.

The application of large‐scale embryo imaging by screening programmes such as DMDD and its counterparts from the International Mouse Phenotyping Consortium (www.mousephenotype.org) is yielding new information both on the nature of mutant phenotypes (Dickinson et al. 2016; Wilson et al. 2016a) and on normal anatomy of the developing embryo. One important consequence of this is the iterative nature of phenotype diagnosis. As the repositories of image data increase and their contents are trawled, our understanding of normal development is being refined and altered. As a result, scoring of phenotypes such as those annotated by DMDD (www.dmdd.org.uk) will inevitably undergo some revision and reassessment.

## Competing interests

No competing interests declared.

## Author contributions

S. H. Geyer collected and analysed data and prepared figures; L. F. Reissig and D. Szumska phenotyped embryos; M. Hüsemann and C. Höfle analysed data; R. Wilson and F. Prin processed and managed image data; A. Galli, D. J. Adams, and J. White identified lethal lines and provided embryos; S. H. Geyer, T. J. Mohun and W. J. Weninger prepared the manuscript.

## Funding

This work was supported by the Wellcome Trust [100160], [FC001157], [FC001117]; Cancer Research UK [FC001157], [FC001117]; and the UK Medical Research Council [FC001157], [FC001117].
